# Advantages of Aseptic Surgery

**Published:** 1899-09

**Authors:** 


In view of what is now known of wound-treatment, it is remarkable that the veterinarian is so slow in adopting aseptic therapy. The fact that human clinical methods are not, as a rule, applicable to veterinary practice is no excuse for not working out methods that can be applied to veterinary operations. The filthy stable, the hairy skin, the dirty unkempt groom, the uncontrollable patient, etc., so often used as an excuse for the abominable disregard for asepsis in vogue in veterinary practice in this country, are by no means insurmountable obstacles. Each of these disadvantages will be overcome when we learn to dispense with our ancient, ante-Lister methods and begin work in accordance with modern knowledge of wound-treatment. Shall we continue to exert ourselves in scientific research if we are not ingenious enough to apply the knowledge thus gained?—*Id.*

